# Prevalence of Antibiotic Resistance in *Salmonella* Serotypes Concurrently Isolated from the Environment, Animals, and Humans in South Africa: A Systematic Review and Meta-Analysis

**DOI:** 10.3390/antibiotics10121435

**Published:** 2021-11-23

**Authors:** Tsepo Ramatla, Mpho Tawana, ThankGod E. Onyiche, Kgaugelo E. Lekota, Oriel Thekisoe

**Affiliations:** 1 Unit for Environmental Sciences and Management, North-West University, Potchefstroom 2531, South Africa; 21491895@student.g.nwu.ac.za (M.T.); Lekota.Lekota@nwu.ac.za (K.E.L.); Oriel.Thekisoe@nwu.ac.za (O.T.); 2 Department of Veterinary Parasitology and Entomology, University of Maiduguri, P.M.B. 1069, Maiduguri 600230, Nigeria; et.onyiche@unimaid.edu.ng

**Keywords:** antibiotic resistance, *Salmonella*, meta-analysis, South Africa

## Abstract

One of the main global concerns is the usage and spread of antibiotic resistant *Salmonella* serovars. The animals, humans, and environmental components interact and contribute to the rapid emergence and spread of antimicrobial resistance, directly or indirectly. Therefore, this study aimed to determine antibiotic resistance (AR) profiles of *Salmonella* serotypes isolated from the environment, animals, and humans in South Africa by a systematic review and meta-analysis. The preferred reporting items for systematic reviews and meta-analyses (PRISMA) guidelines were followed to search four databases for studies published from 1980 to 2021, that reported the antibiotic resistance profiles of *Salmonella* serotypes isolated in South Africa. The AR was screened from 2930 *Salmonella* serotypes which were isolated from 6842 samples. The Western Cape province had high pooled prevalence estimates (PPE) of *Salmonella* isolates with AR profiles followed by North West, Gauteng, and Eastern Cape with 94.3%, 75.4%, 59.4%, and 46.2%, respectively. The high PPE and heterogeneity were observed from environmental samples [69.6 (95% CI: 41.7−88.3), Q = 303.643, I^2^ = 98.353, *Q-P* = 0.045], animals [41.9 (95% CI: 18.5–69.5), Q = 637.355, I^2^ = 98.745, *Q-P* = 0.577], as well as animals/environment [95.9 (95% CI: 5.4−100), Q = 55.253, I^2^ = 96.380, *Q-P* = 0.300]. The majority of the *salmonella* isolates were resistant to sulphonamides (92.0%), enrofloxacin and erythromycin (89.3%), oxytetracycline (77.4%), imipenem (72.6%), tetracycline (67.4%), as well as trimethoprim (52.2%), among the environment, animals, and humans. The level of multidrug-resistance recorded for *Salmonella* isolates was 28.5% in this review. This study has highlighted the occurrence of AR by *Salmonella* isolates from animals, humans, and environmental samples in South Africa and this calls for a consolidated “One Health” approach for antimicrobial resistance epidemiological research, as well as the formulation of necessary intervention measures to prevent further spread.

## 1. Introduction

*Salmonella* is a genus of Gram-negative rod-shaped bacterium of the Enterobacteriaceae family that includes more than 2700 *Salmonella* serotypes [1], found in three species, *Salmonella bongori*, *Salmonella subterranean,* and *S. enterica* [2]. Furthermore, *S. enterica* is divided into six subspecies namely: Indica (subsp. VI), arizonae (subsp. IIIa), houtenae (subsp. IV), enterica (subsp. I), salamae (subsp. II), and diarizonae (subsp. IIIb) [2]. Depending on the serovars and hosts, these bacterial species can cause bacteremia, typhoid fever, gastroenteritis, and non-typhoidal salmonellosis in humans and animals [3,4]. Salmonellosis is one of the most frequent and economically significant zoonotic infections of humans. In addition, it impacts all of the domestic animal species, as well as the wildlife.

Most of the *Salmonella* infections are limited to uncomplicated gastroenteritis that seldom need an antibiotic treatment. However, one of the main global concerns is the usage and spread of drug-resistant *Salmonella* serovars [3]. Different antibiotics have been used to treat *Salmonella* infection in animals and humans [2,5]. Humans, animals, and environmental components interact and contribute to the rapid emergence and spread of antimicrobial resistance, directly or indirectly [6]. Antimicrobial resistance (AMR) among salmonellae has evidently increased, according to surveillance data [2]. One of the causes for the emergence of AR might be due to the use of antimicrobials for metaphylaxis, prophylaxis, treatments, and growth promotion [7]. The common and unregulated use of antibiotics in medical and veterinary practice lead to a continual growth in AMR globally [6]. Regardless of their benefits, the continued release of antibiotics into the environment, as well as their potential negative impact on living organisms, is a major source of concern [8]. 

The development of AR in *Salmonella* strains is generally encoded by mutations in some chromosomal genes and plasmids acquired as a result of antibiotic selective pressure [9]. Resistance plasmids acquired due to the antibiotic selective pressure are commonly used to encode antibiotic resistance in *Salmonella* strains. Horizontal gene transfer plays a role in increasing the spread of AR genes. The use of ciprofloxacin is considered to be the first drug line of choice. However, ciprofloxacin-resistant isolates have been discovered in India [10,11] and Egypt [12], among other countries. This poses a challenge in the public health, especially with different AR genes found in several *Salmonella* serotypes, as shown in a study by El-Sharkawy et al. [12].

In the study conducted by Kalule et al. [13] in South Africa, *Salmonella* serovars isolated from both humans and meat samples demonstrated the existence of several multidrug resistant (MDR) strains to commonly used antibiotics, such as gentamicin and ciprofloxacin. Therefore, a greater knowledge of the possible mechanisms that lead to the emergence of AR in *Salmonella* species should aid in the development of more effective interventional measures, in order to decrease the spread of MDR strains between animals and humans [2]. 

This study includes a comprehensive evaluation of scientific literature published between January 1980 and August 2021 on AR, expressed by *Salmonella* serotypes, isolated from the environment, animals, and humans in South Africa. 

## 2. Results

### 2.1. Overview of the Selected Studies

**Figure 1:**
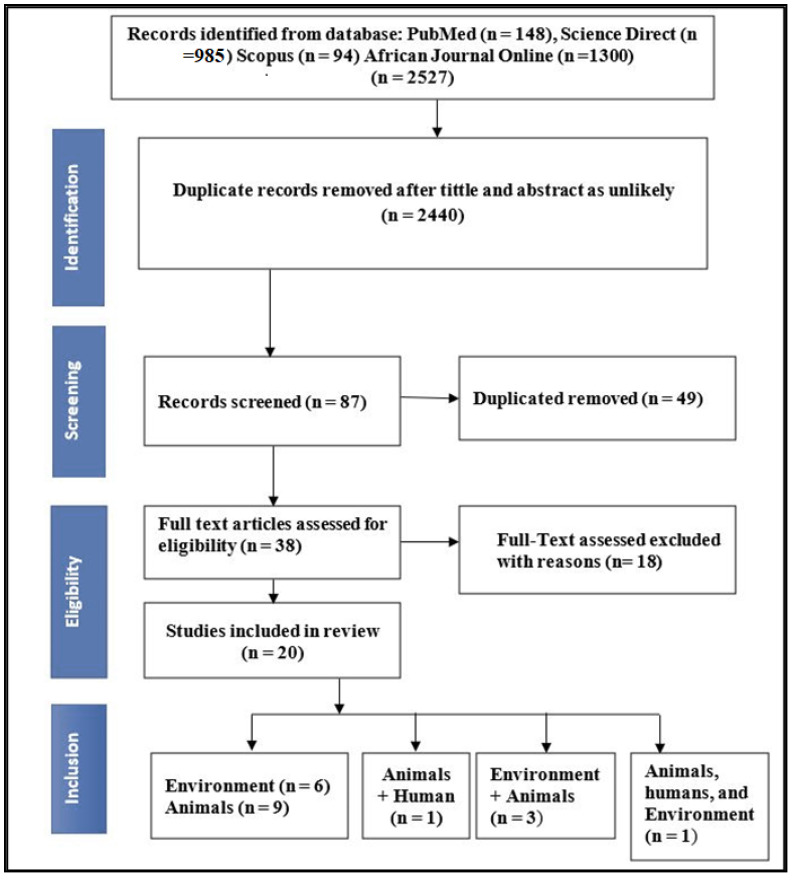
PRISMA flowchart illustrating the process of identifying, screening, and selecting the eligible articles used in this study.

As shown in Figure 1, an electronic search of the databases PubMed, Science Direct, Africa Journals Online, and Scopus yielded 2527 articles. After screening the title and abstract, about 2440 articles were removed. Duplication resulted in the removal of 49 articles. We evaluated 38 full-text papers for eligibility, and 18 papers did not meet our requirements for various reasons: (i) Case studies (*n* = 5) and (ii) studies without clear total samples collected (*n* = 13). Finally, 20 articles with 2930 isolates were included for systemic review and meta-analysis. 

### 2.2. Study Characteristics of Eligible Studies

The following are characteristics of the studies that are eligible: Articles published primarily on the quantitative prevalence of AR by *Salmonella* serovars/isolates in humans, animals (chicken, ducks, cows, pigs, goats, horses, and sheep), and the environment in South Africa; the type of samples and method of diagnostics used; the exact number of samples as well as the number of positives tested; articles reported in English only; and antibiotic resistance. All of the journal papers were published between the years 1980 and 2020.

**Figure 2:**
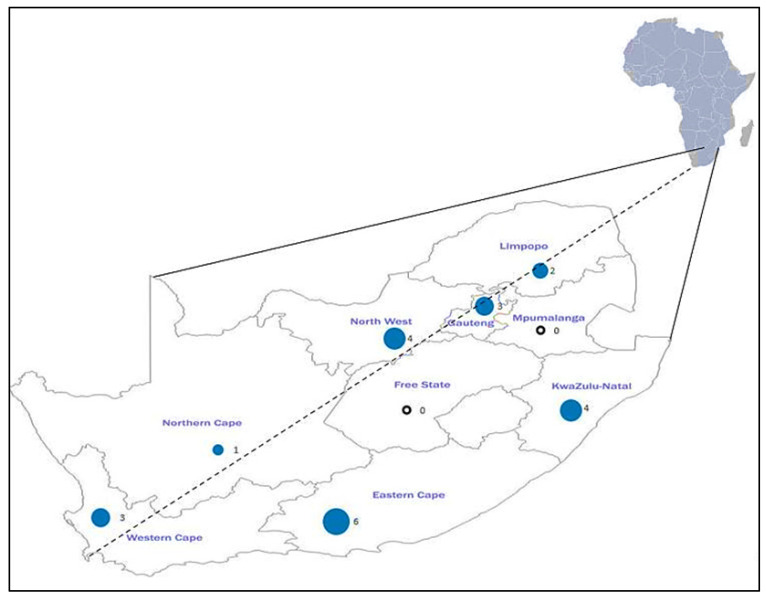
Map showing the number of published studies on *Salmonella* antibiotic resistance per province. The black circle shows that there were no studies conducted.

All of the studies included in this review were derived from all of the provinces in South Africa. Eastern Cape (*n* = 6) was observed to have the highest number of studies followed by North West (*n* = 4), KwaZulu-Natal (*n* = 4), Western Cape (*n* = 3), Gauteng (*n* = 3), Limpopo (*n* = 2), and Northern Cape (*n* = 1) as the least (Figure 2). The most common method for determining AR profiles of *Salmonella* serotypes isolated from all of the studies included in this systematic and meta-analysis study was disk diffusion. Of the 20 included studies, six were environmental samples, nine were samples from animal sources, and three included both animal and environmental samples. One study examined animals, humans, and environmental samples, and one study included samples from both animals and humans.

### 2.3. Pooling and Heterogeneity Estimates of Salmonella Species

#### Prevalence Based on Provinces, Study Years, and Diagnostic Techniques

Table 1 displays a summary of the number of samples processed per subgroup, and the number of those samples that were positive for *Salmonella* serovars. Overall, the assessment of South African data revealed a pooled prevalence estimate (PPE) of 32.3% of processed samples positive for *Salmonella*, with a range between 41.9% and 95.9%. At 95% CI, 30.9–33.9 samples in South Africa would be expected to be positive for *Salmonella* serovars, and the range could be between 5.4% and 100%.

The analyses of the PPE in animals, environment, and animal/environment analyses all revealed a significant heterogeneity. For the environment, pooled heterogeneity was observed [69.6% (95% CI: 41.7–88.3), Q = 303.643, I^2^ = 98.353, *Q-P* = 0.045], animals [41.9% (95% CI: 18.5–69.5), Q = 637.355, I^2^ = 98.745, *Q-P* = 0.577], animals/environment [95.9% (95% CI: 5.4–100), Q = 55.253, I^2^ = 96.380, *Q-P* = 0.300]. Animals/humans/environment, animals/humans, and animal/humans/environment were not included on the meta-analyses due to the low number of studies.

The PPE of *Salmonella* isolates found in Western Cape province was 94.3% (95% CI: 1.1–100), followed by North West, Gauteng, Eastern Cape, and KwaZulu-Natal provinces with 75.4% (95% CI: 19.4–97.5); 59.4% (95% CI: 4.1–98.1); 46.2% (95% CI: 21.5–73.0); and 40.8% (95% CI: 7.4–85.6), respectively. However, Northern Cape and Limpopo were not included on the meta-analyses due to the low number of studies.

A high PPE was observed from the studies conducted during the 2010–2021 period [49.8% (95% CI: 33.8–65.9), Q = 1209.499, I^2^ = 98.512, *Q-P* = 0.981]. Studies conducted between the 2000–2010 period were not included in the meta-analysis since they were less than three. 

*Salmonella* species were identified using three diagnostic techniques, including cultures which showed the greatest degree of sensitivity with PPE of 76.9% (95% CI: 14.6–98.5), Q = 89.158, I^2^ = 95.514, *Q-P* = 0.428, followed by PCR [60.2% (95% CI: 40.9–76.8), Q = 608.599, I^2^ = 98.357, *Q-P* = 0.299], and serotyping [22.4% (95% CI: 3.2–71.3), Q = 42.054, I^2^ = 95.244, *Q-P* = 0.258]. A combination of MALDI-TOF-MS and PCR was not included on the meta-analysis due to the low number of studies. 

### 2.4. Antibiotic Resistance Profile of Salmonella spp. Isolates to Antibiotics

**Figure 3:**
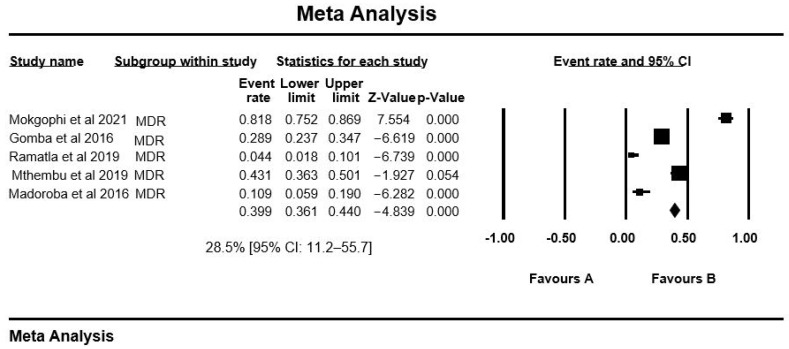
Forest plot showing the pooled estimates of multidrug resistance in studies conducted in South Africa. The diamond at the base indicates the pooled estimates from the overall studies.

The PPE of antibiotic resistance for *Salmonella* serovars included in this meta-analysis (Table 1) was as follows: Sulphonamides with high PPE of 92.0% [95% CI: 37.5–99.5], enofloxacin and erythromycin 89.3% [95% CI: 62.9–97.6], oxytetracycline 77.4% [95% CI: 31.2–96.3], imipenem 72.6% [95% CI: 23.1–95.9], tetracycline 67.4% [95% CI: 53.8–78.6], trimethoprim 52.2% [95% CI: 24.7–78.4], trimethoprim-sulfamthoxazole 47.5% [95% CI: 26.3–69.6], nalidixic acid 39.8% [95% CI: 22.3–60.5], sulphamethoxazole 39.5% [95% CI: 32.6–46.8], ampicillin 38.6% [95% CI: 25.4–53.7], streptomycin 37.7% [95% CI: 17.2–63.8], and the remaining PPE of AR are shown in Table 2. Ampicillin was the most tested antibiotic with 13 studies. Overall, the MDR recorded for *Salmonella* spp. was 28.5% [95% CI: 11.2–55.7]. The overall effect estimates and their accompanying 95% confidence intervals (CI) did not overlap in any of the meta-analyses performed (Figure 3).

The AR was screened from a total of 8843 *Salmonella* serovars, collected from 20 studies. A total of 35 antibiotics were tested on these isolates. The majority of the isolates tested for AR vary by provinces (Table 3), which included Eastern Cape (*n* = 2439), followed by KwaZulu-Natal (*n* = 2094), Limpopo (*n* = 1673), Northern Cape (*n* = 1069), Gauteng (*n* = 793), Western Cape (*n* = 606), and finally by North West (*n* = 169). In terms of the antibiotic profile tested, ampicillin was the most tested antibiotic in all of the provinces. A total of five studies reported multidrug resistance, which is defined as resistance to at least three classes of antibiotics.

## 3. Discussion

In this review, the meta-analysis was used to evaluate the prevalence of AR in *Salmonella* serovars isolated from the environment, animals, and humans. This study examined 20 published articles from South Africa on AR in *Salmonella* serovars. 

The majority (95%) of the studies included in this review were conducted between 2010 to 2021. Based on our meta-analysis, the PPE of AR against sulfonamide was 92%. Our findings correlate with other studies, which reported an increased development of AR against sulfonamide in Spain, Ghana, Central African Republic, Morocco, and Italy with 38.1% 72.4%, 29%, 25%, and 69%, respectively [14,15,16,17,18]. Sulfonamides resistance can arise from chromosomal dihydropteroate synthase (DHPS) mutations or from the acquisition of DHPS drug resistance genes, whose products have lower affinity for sulfonamides [19,20]. The presence of *sul* genes, which encode dihydropteroate synthase in a form that is not inhibited by the drug, is linked to sulfonamide resistance in *Salmonella* serotypes [20]. Generally, sulfonamide is encoded by three *sul* genes which are *sul*1, *sul*2, and *sul*3 [20]. The *sul*1 and *sul*2 genes are the most frequently reported genes found among sulfonamide-resistant isolates, and also found in plasmids of other *Salmonella* species which are still common in Gram-negative bacterial plasmids [19,20,21].

In this meta-analysis, the PPE of tetracycline was 67.4%. Our results are consistent with the findings of the studies conducted in Iran, United States, Saudi Arabia, and China, which reported an increasing development of AR against tetracycline of 66.9%, 63%, 90.71%, and 43%, respectively by *Salmonella* isolates [22,23,24,25]. In South Africa, over 70% of the antibiotics used in livestock production are available over the counter [26], and this contributes to the increased prevalence of AR in the country. Tetracyclines have been an important class of antibiotics in the health and production of food animals for decades, as they are used in veterinary medicine for illness prevention and control [27]. They have a broad spectrum and have good activity against Gram-positive and Gram-negative bacteria that cause acute infections [28]. 

Tetracyclines act as protein synthesis inhibitors by binding to the small ribosomal subunit and preventing aminoacyl-tRNA from attaching to the protein synthesis complex [22]. The complicated syntheses of several systems are capable of conferring tetracycline resistance, including efflux pumps, enzymatic inactivation, and mutations [22,27,29,30]. In South Africa, tetracyclines are the most often used or over-used antibiotics in the livestock production [31]. This is due to the fact that they are relatively affordable and widely available as over-the-counter veterinary medications [22,31,32]. 

According to a survey conducted by Eagar et al. [33], 16.7% and 12.4% of tetracyclines and sulphonamides were commonly used antibiotics in animals in South Africa between the years 2002 and 2004. The Stock Remedies Act of 1947 in South Africa consents tetracycline to be purchased over the counter without a prescription from a veterinarian [26]. 

The PPE of AR for streptomycin by *Salmonella* isolates was 37.7%. Our results are lower than the observations from other studies for resistance against streptomycin in Saudi Arabia, Italy, and Egypt with prevalence of 80%, 95%, 65.5%, and 20.1% [12,34,35,36]. Among the different varieties of aminoglycoside adenylyltransferase coding genes, *aadA* provides resistance to streptomycin in *Salmonella* [37,38]. Streptomycin is one of the most commonly antibiotic used by farmers and veterinarians in the country [31]. The widespread use of antibiotics in the treatment of bacterial infections in plants and animals could explain the high prevalence of streptomycin resistance [26]. 

The current review also revealed that most of the *Salmonella* isolates have high PPE for AR against erythromycin (89.3%). Similar findings were observed previously in studies from Nigeria, Saudi Arabia, Ghana, and South Africa [Gauteng] with 100%, 100%, 86.0%, and 94.9% resistance to erythromycin [31,34,39,40].

Amoxicillin and ampicillins are two of the most common antibiotics used globally to treat salmonellosis [41,42]. In this study, the PPE for AR by *Salmonella* isolates for amoxicillin and ampicillins were lower as compared to the other antibiotics with 19.2% and 38.6%, respectively. Our results were much higher than 0.43% [43] and 0% [44], which were recorded earlier. Antimicrobials, such as ampicillin and others, are still widely used in livestock production in South Africa for growth promotion, prophylaxis, and treatment [26], and in hospitals [43].

The antibiotic imipenem combined with cilastatin is commonly used to prevent its degradation by renal tubular dehydropeptidase. Resistance to this medicine has been reported in a number of bacterial species, adding to the worldwide burden of antibiotic resistance in humans. The PPE of imipenem 72.6% in this study was found to be much higher than the one conducted by Ng and Rivera [45], whereby none of the *Salmonella enterica* isolates were resistant to imipenem.

A total of five studies reported multidrug resistance for the current study. In addition, our results are in line with previous study [46] where multidrug resistance (MDR) was exhibited.

This is the first comprehensive study addressing AR for *Salmonella* serovars isolated from the environment, humans, and animals in South Africa. This study selected peer reviewed and high-quality papers to provide a summary of unbiased findings on the prevalence of AR in *Salmonella* species isolated from the environment, humans, and animals in South Africa. In addition, it reviews the current knowledge and aids in identifying areas where appropriate evidence is lacking, resulting in new research questions/topics. With respect to provinces, there are some provinces where few or no *Salmonella* AR studies were conducted. Between the study periods, there was a greater disparity in study outputs, from one publication in 2000–2010 to 19 articles published between 2010–2021. In terms of overall studies, only two AR studies were conducted on *Salmonella* infecting humans. 

Despite the fact that we have systematized data on the occurrence of AR of *Salmonella* species, this study has the following limitations: The PPE of animals/humans and animals/humans/environment were not calculated since there is a single published article on each. The number of studies from some provinces were unusually high, which may have influenced the overall estimate. Moreover, few reports for *Salmonella* AR from humans were observed, whereby only two studies were undertaken in two provinces, namely, Western Cape and KwaZulu-Natal. Furthermore, the AR genes were not included from this meta-analysis due to the few number of studies conducted.

## 4. Materials and Methods

### 4.1. Study Design

The current systematic review and meta-analysis were conducted in accordance with the preferred reporting items for systematic reviews and meta-analyses (PRISMA 2020) guidelines for selection criteria, literature search, statistical analysis, and data extraction [47].

### 4.2. Search Strategy

The literature search was performed on four databases, namely, (https://www.ncbi.nlm.nih.gov/pubmed, accessed on 27–28 May 2021), ScienceDirect (https://www.sciencedirect.com, accessed on 5–7 June 2021), African Journal Online (https://www.ajol.info/index.php/ajol, accessed on 11–12 June 2021), and Scopus (https://www.scopus.com, accessed on 22–24 June 2021) from June to August 2021. The following keywords were used to search for the articles: Antibiotic resistance AND Antibiotic AND drug resistance AND bacteria resistance AND multi-drug resistance AND *Salmonella* species AND Human OR animal AND Environment AND salmonellosis AND South Africa. We conducted our last search on 24 June 2021. 

### 4.3. Inclusion and Exclusion Criteria

The following inclusion criteria had to be met for all of the eligible studies included in the review: Articles published primarily on the quantitative prevalence of *Salmonella* spp. in the environment, animals, and humans in South Africa; the type of samples and method of diagnostics used; the exact number of samples as well as the number of positives tested; and articles reported in English only on antibiotic resistance, published between January 1980 and August 2021. The studies were excluded if they were not undertaken in South Africa (Supplementary Materials). In addition, books and book chapters were excluded. Moreover, a review, a smaller sample size, and articles not reported in English, were discarded, as well as articles not published between January 1980 and August 2021.

### 4.4. Data extraction and Data Collection

The titles and abstracts of journal articles were examined and downloaded, and the full versions of potentially relevant articles were obtained to determine eligibility. Data including names of authors, publication year, location, total sample size, and standard methods to detect the antibiotic resistant were collected from each publication independently. Then, they were entered into a spreadsheet (Microsoft Excel^®^), tables, and a word document template. Only *Salmonella* species/isolates/serotypes specific journal articles were included in the meta-analysis.

The studies with insufficient data were excluded. In addition, review articles and studies with an abstract, but without a full text, were excluded. The papers that included the first author, year of publication, year of study, sampling, region, sample size, target population, method, drug susceptibility test, sample type, number of isolates, and number of multidrug resistance strains were considered and included in this study. For inclusion in the study, we considered all of the standard guidelines, although only the clinical and laboratory standards institute (CLSI) guidelines were used in all of the included studies (Table 4).

### 4.5. Data Analysis and Assessment of Risk of Bias

The Begg’s and Egger’s tests were used to investigate the possibility of propagation bias. For each study, the prevalence, effect size, and 95% CIs were calculated as a point estimate. 

A comprehensive meta-analysis was used for all of the statistical analyses, version 3.0 (CMA) program (https://www.meta-analysis.com/, accessed on 18 August 2021). The software was used to generate the pooled estimates, Cochran’s Q, *p*-values, and forest plots. I^2^ (level of inconsistency) was used to assess the study heterogeneity (Cochran’s Q). The I^2^ values above 75% were regarded to have a high degree of heterogeneity [64]. For each study, the prevalence, effect size, and 95% CIs were calculated as a point estimate. Exploring the funnel plots was used to assess the publication bias. 

## 5. Conclusions

This systematic review and meta-analysis provided an overview of published *Salmonella* serovar AR scientific data from humans, animals, and environmental samples in South Africa. The higher PPE rate of *Salmonella* isolates were tested. In addition, expressing AR was observed in the Western Cape province. Disk diffusion was the most prevalent method for identifying the antibiotic resistance profiles of *Salmonella* serotypes isolated from all of the studies. The MDR has been identified as a serious and growing issue, according to the findings of this study. Our results revealed the highest PPE of *Salmonella* AR to sulphonamides, followed by enrofloxacin and erythromycin. In addition, this finding calls for the restricted usage of this group of antibiotics. According to our data analysis, the most tested antibiotics against *Salmonella* isolates are tetracycline, ciprofloxacin, chloramphenicol, ampicillin, streptomycin, gentamicin, erythromycin, and kanamycin. This study highlights the lack of published *Salmonella* serovar AR scientific data from humans. The development of AR to commonly prescribed antibiotics is very common, whilst it appears that surveillance has a lot of gaps whereby there has been no studies on AR in two provinces, namely, Free State and Mpumalanga. To stop the spread of *Salmonella* AR, control strategies should be strengthened. To describe the epidemiology of the serotypes across the country, large-scale investigations are required. Control practices should be strengthened to slow the spread of AR in South Africa and there is a need for a “One Health” collaborative research between the animal and human health sector, as well as the environmental sector on the epidemiology of AR development and necessary interventions.

## Figures and Tables

**Table 1:** Pooled prevalence of *Salmonella* spp. from the environment, animals, and humans; screening methods; study year; and sampling sites.

Risk Factors	Number of Studies	Pooled Estimates					Measure of Heterogeneity		
		Sample Size	Number Positive	Pooled Prevalence	I^2^ (95% CI)	Cochran’s Q	Heterogeneity I^2^ (%)	Significance Level *Q-p*	*p*-Value
Overall study									
Environment	6	1801	942	69.9	(41.7–88.3)	303.643	98.353	0.161	0.04544
Animals	9	3722	1000	41.9	(18.5–69.5)	637.355	98.745	0.577	0.33833
Animals/humans	1	200	146	-	-	-		-	-
Animals/environment	3	904	834	95.9	(5.4–100)	55.253	96.380	0.304	0.30075
Animals/humans/environment	1	215	8	-	-	-		-	-
Study year									
2000–2010	1	172	172	-	-	-		-	-
2010–2021	19	6691	2551	49.8	(33.8–65.9)	1209.499	98.512	0.981	0.29987
Diagnostic technique									
PCR	11	3475	1551	60.2	(40.9–76.8)	608.599	98.357	0.299	0.46897
Culture	5	1463	778	76.9	(14.6–98.5)	89.158	95.514	0.428	0.16359
Serotype	3	1434	338	22.4	(3.2–71.3)	42.054	95.244	0.258	0.05859
MALDI-TOF-MS and PCR	1	481	263	-	-	-		-	-
Provinces									
KwaZulu-Natal	4	2094	470	40.8	(7.4–85.6)	404.768	99.259	0.735	0.50000
Gauteng	3	793	436	59.4	(4.1–98.1)	62.831	96.817	0.832	0.30075
Eastern Cape	6	2439	785	46.2	(21.5–73.0)	381.864	98.691	0.796	0.09424
North West	4	169	528	75.4	(19.4–97.5)	250.522	98.802	0.389	0.24845
Northern Cape	1	1069	30	-	-	-		-	-
Limpopo	2	1673	122	-	-	-		-	-
Western Cape	3	606	685	94.3	(1.1–100)	75.412	97.348	0.450	0.30075

PCR = Polymerase chain reaction.

**Table 2:** Pooled prevalence rate and 95% CI of antibiotic resistance of *Salmonella* species based on the meta-analysis.

Antimicrobial Agents	Number of Studies	Number of Isolates	% Prevalence (95% CI)	I^2^ (95% CI)
Tetracycline	9	1192	67.4	(53.8–78.6)
Chloramphenicol	8	243	2.6	(14.6–28.2)
Ciprofloxacin	6	167	28.9	(8.5–63.9)
Sulphonamides	3	285	92.0	(37.5–99.5)
Nalidixic acid	3	144	39.8	(22.3–60.5)
Streptomycin	9	593	37.7	(17.2–63.8)
Ampicillin	13	900	38.6	(25.4–53.7)
Streptomycin	9	593	37.7	(17.2–63.8)
Amoxicillin	3	80	19.2	(13.8–26.1)
Trimethoprim	3	477	52.2	(24.7–78.4)
Enrofloxacin	6	20	89.3	(62.9–97.6)
Erythromycin	6	954	89.3	(62.9–97.6)
Gentamicin	6	95	15.4	(7.0–3.5)
Sulphamethoxazole	3	165	39.5	(32.6–46.8)
kanamycin	6	172	26.7	(10.1–54.1)
Imipenem	3	517	72.6	(23.1–95.9)
Oxytetracycline	4	382	77.4	(31.2–96.3)
Trimethoprim-sulfamthoxazole	4	110	47.5	(26.3–69.6)
MDR	5	314	28.5	(11.2–55.7)

**Table 3:** Summary of *Salmonella* spp. resistant isolates of the most tested antibiotics.

Number of Resistant Isolates (%)								
Provinces	Tetracycline	Ciprofloxacin	Chloramphenicol	Ampicillin	Streptomycin	Gentamicin	Erythromycin	Kanamycin
KwaZulu-Natal	52/435: (57.9%)	16/195: (8.2%)	32/146: (21.9%)	257/371: (69.2%)	40/346: (11.5%)	49/146: (33.5%)	18/146: (12.3%)	5/263: (1.9%)
Gauteng	-	1/170: (1.4%)	41/433: (9.4%)	4/263: (1.5%)	141/433: (3.2%)	-	170/170: (100%)	75/146: (51.3%)
Eastern Cape	543/1197: (45.3%)	33/307: (10.7%)	98/370: (26.4%)	487/635: (76.6%)	286/410: (69.7%)	13/112: (11.6%)	406/438: (92.6%)	47/152: (30.9%)
North West	78/114: (68.4%)	125/198: (63.1%)	58/198: (29.2%)	277/498: (45.5%)	73/198: (36.8%)	9/198: (4.5%)	360/384: (93.7%)	-
Northern Cape	-	-	-	5/30: (16.6%)	-	-	-	-
Limpopo	-	-	-	41/122: (33.6%)	-	-	-	27/92: (29.3%)
Western Cape	-	-	-	-	-	2/8: (25%)	-	-

**Table 4:** Studies included in this review, as well as provinces, methods, isolation sources, and *Salmonella* spp./isolates that were tested for antibiotic resistance.

Study (Citation)	Province	Method	Sample Size	No. of Isolates	Isolate Source	*Salmonella* spp.
Gouws et al., 2000 [48]	Western Cape	DDM	Culture	442	Animal/environment	*Salmonella* spp.
Mokgophi et al., 2021 [31]	Gauteng	DDM	PCR	170	Animal	*S*. Bovismorbificans (58.5%); *S*. Dublin (18.5%); *S*. Enteritidis (15.7%); *S*. Mbandaka (12.8%); *S*. Saintpaul (8.5%); *S*. Thompson (2.8%); *S*. Infantis (2.8%); and *S*. Agona (1.4%).
Gomba et al., 2016 [49]	Gauteng	DDM	MALTI-TOF-MS and PCR	263	Environment	*S.* Muenchen (33.3%); *S*. Typhimurium (12/39; 30.8%); *S.* Heidelberg (20.5%); *S.* Bsilla (7.7%).
Ramatla et al., 2019 [50]	North West	DDM	PCR	114	Animal	*S*. Typhimurium (*n* = 44, 30.5%); S. Enteritidis (*n* = 18, 12.5%); *S*. newport (7.6%); *S*. Heidelberg (11.1%); *S. bongori* (9%); *S. enterica* serovar Paratyphi B (4.8%); *S*. Tennessee (2%); and *S*. Pullorum (1.3%).
Adesiyun et al., 2020 [51]	Gauteng	DDM	Culture	3	Animal	*Salmonella* spp.
Mafu et al., 2012 [52]	Eastern Cape	DDM	Culture	40	Environment	*Salmonella* spp.
Jaja et al., 2019 [26]	Eastern Cape	DDM	PCR	112	Animal	*S*. Enteritidis
Mthembu et al., 2019 [53]	Eastern Cape and KwaZulu-Natal	DDM	PCR	194	Environment	*Salmonella* spp.
Iwu et al., 2016 [54]	Eastern Cape	DDM	PCR	258	Animal	*Salmonella* spp.
Akinola et al., 2019 [55]	North West	DDM	PCR	84	Animal	*S*. *bongori* (10.09%); *S.* Pullorum (1.81%); *S.* Typhimurium (12.72%); *S.* Weltevreden; *S.* Chingola; *S.* Houten; and *S.* Bareily (1.81%).
Mathole et al., 2017 [56]	Limpopo, Eastern Cape, Northern Cape, North West, and KwaZulu Natal	DDM	Serotyping	30	Animal	*S*. Chester (3.3%); *S*. Cardoner (3.3%); *S*. Sambrae (3.3%); *S*. Typhimurium (3.3%); *S*. Schwarzengrund (6.6%); *S*. Aarhus (3.3%); *S*. Pomona (33%); *S*. Senftenberg (3.3%); and *S*. Techimani (30%); unclassified *Salmonella* (20%).
Igbinosa 2015 [57]	Eastern Cape	DMD	PCR	150	Environment	*Salmonella* spp.
Odjadjare and Olaniran 2015 [58]	KwaZulu Natal	DMD	PCR	200	Environment	*Salmonella* spp.
Zishiri et al., 2016 [42]	KwaZulu Natal	DMD	PCR	146	Animal/human	*Salmonella* spp.
Madoroba et al., 2016 [59]	Limpopo	DDM	PCR	92	Animal/environment	*S*. Heidelberg (2.2%); *S*. Aberdeen (1.1%); *S*. Hayindongo (1.1%); *S*. Mbandaka (2.2%); *S*. Anatum (2.2%); *S*. Othmarschen (1.1%); S. Nigeria (2.2%); *S*. Tennessee (1.1%); S. Cardoner (1.1%); *S*. Senftenberg (2.2%); and *S*. Pretoria (2.2%).
More et al., 2017 [60]	Western Cape	DDM	Culture	235	Animal	*Salmonella* spp.
Kennedy et al., 2020 [61]	KwaZulu Natal	DDM	PCR	94	Environment	*Salmonella* spp.
Dlamini et al., 2018 [62]	North West	DDM	Serotyping	300	Animal/environment	*Salmonella* spp.
Kalule et al., 2019 [13]	Western Cape	DDM	Serotyping	8	Human/animal/environment	*S*. *enterica*
Chipangura et al., 2017 [63]	South Africa	DDM	Culture	58	Animal	*Salmonella* spp.

DDM: Disc-diffusion method; PCR = polymerase chain reaction.

## Data Availability

The data presented in this study are available on request from the corresponding author.

## Supplementary Materials

The following are available online at https://www.mdpi.com/article/10.3390/antibiotics10121435/s1, Table S1: Checklist of items to include when reporting a systematic review or meta-analysis, Figure S1: Forest plot of the meta-analysis of overall studies on the prevalence of Salmonella isolates in South Africa.
