# Supplementary material for: Prevalence of Antibiotic Resistance in Salmonella Serotypes Concurrently Isolated from the Environment, Animals, and Humans in South Africa: A Systematic Review and Meta-Analysis
Source: Antibiotics (Basel). 2021 Nov 23;10(12):1435. doi: 10.3390/antibiotics10121435 (PMC8698067; doi:10.3390/antibiotics10121435)
Supplement: Supplementary file 1 [file antibiotics-10-01435-s001.zip › antibiotics-1462501-supplementary.pdf]

Supplementary Materials

Table S1. Checklist of items to include when reporting a systematic review or meta-analysis

Section/topic	#	Checklist item	Reported on page #
**TITLE**			
Title	1	Identify the report as a systematic review, meta-analysis, or both.	1
**ABSTRACT**			
Structured summary	2	Provide a structured summary including, as applicable: background; objectives; data sources; study eligibility criteria, participants, and interventions; study appraisal and synthesis methods; results; limitations; conclusions and implications of key findings; systematic review registration number.	2
**INTRODUCTION**			
Rationale	3	Describe the rationale for the review in the context of what is already known.	2-4
Objectives	4	Provide an explicit statement of questions being addressed with reference to participants, interventions, comparisons, outcomes, and study design (PICOS).	2-4
**METHODS**			
Protocol and registration	5	Indicate if a review protocol exists, if and where it can be accessed (e.g., Web address), and, if available, provide registration information including registration number.	-
Eligibility criteria	6	Specify study characteristics (e.g., PICOS, length of follow-up) and report characteristics (e.g., years considered, language, publication status) used as criteria for eligibility, giving rationale.	4
Information sources	7	Describe all information sources (e.g., databases with dates of coverage, contact with study authors to identify additional studies) in the search and date last searched.	4
Search	8	Present full electronic search strategy for at least one database, including any limits used, such that it could be repeated.	4 and Table 1

Section/topic	#	Checklist item	Reported on page #
Study selection	9	State the process for selecting studies (i.e., screening, eligibility, included in systematic review, and, if applicable, included in the meta-analysis).	4-5
Data collection process	10	Describe method of data extraction from reports (e.g., piloted forms, independently, in duplicate) and any processes for obtaining and confirming data from investigators.	4-5
Data items	11	List and define all variables for which data were sought (e.g., PICOS, funding sources) and any assumptions and simplifications made.	4-5
Risk of bias in individual studies	12	Describe methods used for assessing risk of bias of individual studies (including specification of whether this was done at the study or outcome level), and how this information is to be used in any data synthesis.	4-5
Summary measures	13	State the principal summary measures (e.g., risk ratio, difference in means).	4-5
Synthesis of results	14	Describe the methods of handling data and combining results of studies, if done, including measures of consistency (e.g., I^2) for each meta-analysis.	4-5
Risk of bias across studies	15	Specify any assessment of risk of bias that may affect the cumulative evidence (e.g., publication bias, selective reporting within studies).	4-5
Additional analyses	16	Describe methods of additional analyses (e.g., sensitivity or subgroup analyses, meta-regression), if done, indicating which were pre-specified.	5-6
**RESULTS**			
Study selection	17	Give numbers of studies screened, assessed for eligibility, and included in the review, with reasons for exclusions at each stage, ideally with a flow diagram.	6
Study characteristics	18	For each study, present characteristics for which data were extracted (e.g., study size, PICOS, follow-up period) and provide the citations.	Table 2
Risk of bias within studies	19	Present data on risk of bias of each study and, if available, any outcome-level assessment (see Item 12).	S1 file
Results of individual studies	20	For all outcomes considered (benefits or harms), present, for each study: (a) simple summary data for each intervention group and (b) effect estimates and	Tables 3-5, Figures 3-4

Section/topic	#	Checklist item	Reported on page #
		confidence intervals, ideally with a forest plot.	
Synthesis of results	21	Present results of each meta-analysis done, including confidence intervals and measures of consistency.	6-12
Risk of bias across studies	22	Present results of any assessment of risk of bias across studies (see Item 15).	12
Additional analysis	23	Give results of additional analyses, if done (e.g., sensitivity or subgroup analyses, meta-regression) (see Item 16).	
**DISCUSSION**			
Summary of evidence	24	Summarize the main findings including the strength of evidence for each main outcome; consider their relevance to key groups (e.g., health care providers, users, and policy makers).	12-16
Limitations	25	Discuss limitations at study and outcome level (e.g., risk of bias), and at review level (e.g., incomplete retrieval of identified research, reporting bias).	16
Conclusions	26	Provide a general interpretation of the results in the context of other evidence, and implications for future research.	16
**FUNDING**			
Funding	27	Describe sources of funding for the systematic review and other support (e.g., supply of data); role of funders for the systematic review.	17

Meta Analysis

Study name	Event rate	Lower limit	Upper limit	Z-Value	p-Value
Gouws et al 2000	0,997	0,956	1,000	4,126	0,000
Mokgophi et al 2021	0,997	0,950	1,000	4,034	0,000
Gomba et al 2016	0,536	0,491	0,579	1,578	0,115
Ramatla et al 2019	0,416	0,359	0,475	-2,766	0,006
Adesiyun et al 2020	0,020	0,006	0,060	-6,685	0,000
Mafu et al 2012	0,900	0,326	0,994	1,474	0,140
Jaja et al 2019	0,291	0,248	0,338	-7,940	0,000
Mthembu et al 2019	0,540	0,489	0,591	1,525	0,127
Iwu et al 2016	0,516	0,472	0,560	0,715	0,474
Akinola et al 2019	0,991	0,873	0,999	3,315	0,001
Mathole et al 2017	0,028	0,020	0,040	-19,141	0,000
Igbinosa, 2015	0,996	0,937	1,000	3,870	0,000
Odjadjare and Olaniran 2015	0,990	0,857	0,999	3,218	0,001
Zishiri et al 2016	0,730	0,664	0,787	6,245	0,000
Madoroba et al 2016	0,152	0,126	0,183	-15,159	0,000
More et al 2017	0,998	0,966	1,000	4,329	0,000
Kennedy et al 2020	0,121	0,100	0,146	-18,027	0,000
Dlamini et al 2018	0,997	0,949	1,000	4,029	0,000
Kalule et al 2019	0,037	0,019	0,073	-9,029	0,000
Chipangura et al 2017	0,064	0,050	0,082	-19,774	0,000
	0,323	0,308	0,339	-20,671	0,000

32.3% (95% CI: 33.9-30.9)

Event rate and 95% CI

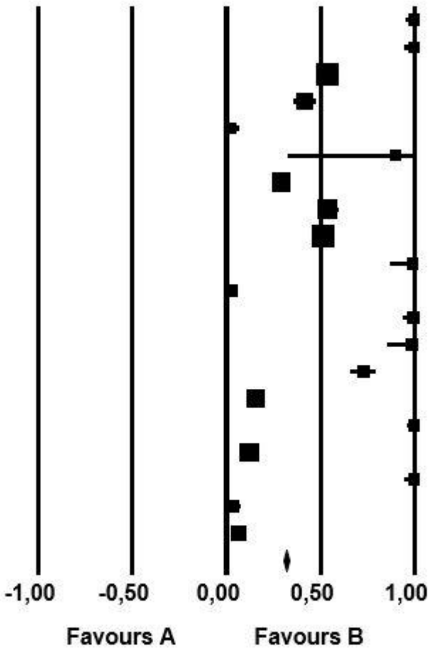

Meta Analysis

Figure S1. Forest plot of the meta-analysis of overall studies on the prevalence of *Salmonella* isolates in South Africa.
